# The Prevalence of Headache and Associated Factors in Al-Kharj, Saudi Arabia: A Cross-Sectional Study

**DOI:** 10.1155/2021/6682094

**Published:** 2021-03-04

**Authors:** Daifallah Almalki, Mamdouh M. Shubair, Badr F Al-Khateeb, Rawan Abdullah Obaid Alshammari, Saeed Mastour Alshahrani, Raed Aldahash, Khadijah Angawi, Majid Alsalamah, Jamaan Al-Zahrani, Sameer Al-Ghamdi, Hayat Saleh Al-Zahrani, Ashraf El-Metwally, Khaled K. Aldossari

**Affiliations:** ^1^Department of Medicine, College of Medicine, Prince Sattam Bin Abdulaziz University, Al-Kharj, Saudi Arabia; ^2^School of Health Sciences, University of Northern British Columbia (UNBC), 3333 University Way, Prince George, British Columbia, Canada; ^3^Department of Family Medicine, King Abdulaziz Medical City, College of Public Health and Health Informatics, King Saud Bin Abdulaziz University for Health Sciences, Riyadh, Saudi Arabia; ^4^Princess Nourah University, Leader of Epidemiology of Batch 39, Member of Quality Club of Health and Rehabilitation Sciences College at Princess Noura University, Riyadh, Saudi Arabia; ^5^College of Applied Medical Sciences, King Khalid University, Abha, Saudi Arabia; ^6^College of Medicine, Medicine Department, King Saud Bin Abdulaziz University for Health Sciences (KSAU-HS), King Abdulaziz Medical City-National Guard, Riyadh, Saudi Arabia; ^7^Department of Health Services and Hospital Administration, Faculty of Economics and Administration, King Abdulaziz University, Jeddah 80200, Saudi Arabia; ^8^Department of Emergency Medicine, College of Medicine, King Saud Bin Abdulaziz University for Health Sciences, Riyadh, Saudi Arabia; ^9^Family & Community Medicine Department, College of Medicine, Prince Sattam Bin Abdulaziz University, Al-Kharj, Saudi Arabia; ^10^Family Medicine and Medical Education, Princess Nourah Bint Abdulrahman University, Riyadh, Saudi Arabia; ^11^College of Public Health and Health Informatics, King Saud Bin Abdulaziz University for Health Sciences, Riyadh, Saudi Arabia

## Abstract

**Background:**

Only few studies have investigated the prevalence and risk factors of headaches among the Saudi population. The study aimed to estimate the prevalence of headache and to explore its associated risk factors Al-Kharj, Saudi Arabia.

**Methods:**

The multistage sampling technique was used to enroll 1200 population-based participants who were asked to complete a self-administered questionnaire about headaches, demographics, and several other parameters such as smoking status and different chronic and psychological illnesses. The chi-square test and multivariate logistic regression analysis were used to test the association.

**Results:**

The overall prevalence of headaches in this study was 3%. The multiple logistic regression analysis showed that females were more likely to have headaches than males (odds ratio (OR) 0.735, 95% confidence interval (CI) = 0.612–1.341; *P*=0.024). Being a current smoker was also significantly associated with higher “odds” of having headache (OR = 1.319, 95% CI = 0.932–2.462; *P*=0.037). Participants who were overweight had a significantly higher risk of headache (OR = 1.631, 95% CI = 1.48–1.854; *P*=0.037). Nonmarried people were significantly more likely to have headache pain, compared to married individuals (OR = 0.875, 95% CI = 0.646–2.317; *P*=0.047).

**Conclusion:**

The prevalence of headaches was 3%, and four significant associated factors were identified: females, nonmarried, smoking, and overweight. The temporality of the relationship between these factors and headache cannot be confirmed in this cross-sectional study; so future longitudinal studies are needed to confirm these potential causal relationships.

## 1. Introduction

Globally, headaches are the significant disorders of the nervous system; almost fifty percent of the adult population suffers from headaches at least once a year [1]. Headache or cephalgia can be defined as a symptom that refers to any pain located in the head or neck. Broadly speaking, there are many types, but they can be divided into two major categories: primary and secondary headaches [2]. Primary headaches account for more than ninety percent of the total headaches, and they are accompanied with painful and disabling characteristics such as tension and cluster headaches [1, 2]. Secondary headaches occur due to underlying chronic conditions and are commonly caused by overuses of medicine [1].

A 2016 study derived from Global Burden of Diseases (GBD), Injuries, and Risk Factors estimated that three billion peoples suffered from primary headaches. Headaches were most prevalent among young females aged 15–49 years, with 11.2% of all years of life lived with disability (YDLs) [3]. However, there was a documented small increase of age-standardized disability adjusted life years with the sociodemographic index [3].

According to Lifting the Burden (the global campaign to reduce the burden of headache worldwide), epidemiological research studies have shown an increased prevalence of headache pain, which include migraines, tension-type headaches, and headaches resulting from overuse of medications, and their relationship with poor quality of life and significant loss of productivity while causing high economic costs in each country was surveyed. A recent GBD study indicates that headaches are the second main reason of years of worldwide disability; whereas, migraine alone is the third largest cause among those who aged 15–49 [4].

A common neurological condition accounted for 1–4 percent of the general population is a chronic daily headache (CDH). Most CDH patients initially suffer from episodic headaches, but after some time, this progressed into CDH. Timely identification of the associated risk factors can reduce this complication and can be targeted with preventive measures [5]. Although CDH's pathophysiology is not fully grasped, recent data suggested insights into risk factors associated with an increased risk of episodic headaches transformation [5].

Frequent headaches are predisposed by certain environmental or genetic determinants. These determinants include but are not limited to excessive intake of caffeine, age, gender, genetic disabilities, overuse of medication, psychiatric comorbidities (anxiety, insomnia, and depression), obesity, temporomandibular disorders, increased susceptibility to chronic headaches, and life events [5–8]. Several studies have shown that chronic dehydration can also be a primary risk factor for headaches. Sleep deprivation (and heavy sleep) can result in severe and chronic headaches, and this can be prevented by adequate sleep between 7 and 9 hours every night [9]. While, reducing the intake of histamine-rich foods (cheese and other fermented foods) can be helpful for people who are predisposed to chronic headaches [10]. Sweet pea essential oil and lavender tea can reduce headache symptoms caused by tension or migraine headaches [11].

Different studies published the prevalence of migraines, and other aspects have also emerged from the Middle Eastern countries; nonetheless, the quantization of these findings has not yet been accomplished sufficiently. Up to date, there are no studies carried out in the general population in Saudi Arabia about the prevalence and associated factors with headache [12]. It is also not clear what factors predict this condition in Saudi Arabia, which leads to the importance of making a prevention plan. Therefore, the study aimed to estimate the prevalence and to explore the associated risk factors of headache in the Al-Kharj population.

## 2. Materials and Methods

This is a population-based cross-sectional study. The study was conducted in the city of Al-Kharj, Saudi Arabia, from January to June in the year 2016. The city was located 77 km south of Riyadh, with a diverse, multiracial, and ethnic population of approximately 376,000. A total of 1019 study participants were included in the study. Inclusion criteria include Saudi national, age ≥18 years, having no terminal illness or mental illness, and willing to participate in the survey.

In contrast, exclusion criteria include non-Saudis, age <18 years, individuals who do not understand the consent form, and are unwilling to participate in the study. A multistage stratified cluster sampling technique was employed for the collection of study data. Participants were invited from different corporate or educational institutions (total 32). These were then divide classified into strata government or private institutes. Cluster sampling was then employed on the selected institutes. Sampling units are then carefully chosen through simple random sampling using the list of employees obtained from each institute's department. The computer-generated list was utilized for the selection of the participants.

The confidentiality of the data was maintained at each level and was available only to the principal investigator. Code was assigned to each respondent, and the anonymous data were given to the researchers. A total of 1200 participants' data were collected, of which 1019 were included in the final analysis after removing the missing variables for this study. The total response rate was 85%.

Data were collected using a predesigned, structured, self-administered questionnaire, which comprised of more than a few sections. The first section comprised sociodemographic information, including age, education, employment, and marital status. The second section included smoking status, headache, and different chronic and psychological illnesses. The third section contained a validated and reliable General Health Questionnaire (GHQ-12) to assess the individuals' psychological distress [13]. Followed by, general physical examination was performed by trained status. Headache was defined as headaches that last for 15 days or more in a month [1].

SPSS version 26 (SPSS Inc., Chicago, Illinois, USA) for Windows was used for data analysis. The chi-square test, bivariate correlation coefficient ((Pearson's *r*), and multivariate logistic regression analysis were used to test the association between headache and different variables. A *P* value of <0.05 was set for statistical significance.

### 2.1. Ethical Consideration

Ethical approval was obtained from the local Committee of Scientific Research and Publications, Prince Sattam Bin Abdulaziz University, Al-Kharj, Saudi Arabia. The IRB number for the study is PSAU/COM/RC/IRB/P/49. The detail about the study procedure was given to all participants, and after their approval, both verbal and written informed consent was obtained. Participation in the study was completely voluntary.

## 3. Results

### 3.1. Univariate Analysis

There was a significant inverse moderate association between headache pain and total GHQ score. Individuals who reported “yes” (for headache pain) had a higher total GHQ score (meaning more psychological distress) compared to individuals who reported “no” (for headache pain). The latter group of individuals had a lower total GHQ score (*r* = −0.095, *P*=0.003).

The prevalence of headache pain as a binary variable (0 = yes, headache pain; 1 = no headache pain) was contrasted with other variables. Nine cross-tabulations (*X*^*2*^) were conducted to explore the association between the presence of headache pain and each of the variables: respondent gender, marital status, education level attainment, job type, prehypertension; hypertension; diabetes, smoking status, and body mass index (BMI) class/category (Table 1).

The females (60.0%) have a significantly higher prevalence of headache pain than the male counterparts (*X*^2^ = 13.090, *P*=0.047). Nonmarried persons (66.7%) were significantly more likely to have headache pain than married individuals (*X*^2^ = 14.039, *P*=0.038). Regarding job type/status, there was no significant statistical association between those unemployed, civilian, or soldier workers (*X*^2^ = 0.620, *P*=0.733). There was no significant difference, whether being prehypertensive or non-prehypertensive, with regards to the prevalence of headache pain. The chi-squared test (*X*^2^) = 1.034, *P*=0.309 (Table 1).

The association between headache pain and hypertension showed a significant increase in the proportion of hypertensive individuals (6.7%) who reported having headache compared with hypertensive people who did not have a headache (4.8%). Notably, a very large proportion (93.3%) of the normotensive persons reported having a headache, compared with 95.2% of normotensive persons who did not have a headache. The chi-squared test (*X*^2^) = 9.221, *P*=0.048. Diabetic status (nondiabetic vs. diabetic individuals) did not show any notable significant differences regarding the prevalence of headache pain. The chi-squared test (*X*^2^) = 1.428, *P*=0.232. The proportion of current smokers who have headache pain (16.7%) was significantly higher than their current smokers' counterparts who do not have a headache (9.7%). This indicates that smoking is associated with an increased probability of having a headache (*X*^2^ = 18.412, *P*=0.027). The results showed that the proportion of overweight individuals who have headache pain is significantly higher (36.7%) than their overweight peers who do not have headache pain (26.4%). The class I obese and class II/III obese categories did not show this same trend (*X*^2^ = 18.570, *P*=0.046) (Table 1).

A first multiple logistic regression analysis was conducted by using the binary headache status (outcome variable) with “raw” (continuous) BMI (independent variable) and other sociodemographic and lifestyle variables (Table 2). The results showed that BMI was positively and significantly associated with the prevalence of headache. The odds ratio (OR) was 1.58 (95% CI = 1.421–1.752; *P*=0.042). Gender status showed a significant inverse association with headache, in that females were more likely to have headache compared to males. The OR was 0.735 (95% CI = 0.612–1.341; *P*=0.024). Being a current smoker was also significantly associated with higher “odds” (1.32 times) of having headache (OR = 1.319, 95% CI = 0.932–2.462; *P*=0.037), as shown in Table 2.

A second multiple logistic regression analysis was further performed to examine the association between BMI class (categorical variable) and the relative risk of headache after adjusting for sociodemographic and other lifestyle variables (Table 3). The results showed that overweight respondents had a significantly higher risk of headache. The odds ratio (OR) was 1.631 (95% CI = 1.48–1.854; *P*=0.037). Females were significantly more likely to have a higher prevalence of headache (OR = 0.604, 95% CI = 0.417–1.283; *P*=0.023), compared to males. In regards to marital status, nonmarried people were significantly more likely to have headache pain, compared to married individuals (OR = 0.875, 95% CI = 0.646–2.317; *P*=0.047), as illustrated in Table 3.

## 4. Discussion

This cross-sectional study investigated the prevalence of headaches and associated risk factors in the participants of city of Al-Kharj. The study noted an increased prevalence of headaches, with a significant increase in the odds of having headaches among female participants who were overweight, current smoker, and nonmarried. The prevalence of headaches in our study was 3%, consistent with the WHO and headache classification committee [1].

The multiple logistic regression analysis in this study showed that females were more likely of developing headaches as compared to males. This is similar to a study published in 2019 that found migraine prevalence to be twice as high in females than males [14]. Furthermore, a study in India reported the prevalence of headaches was three times as high in females (8.8%) than males (2.9%) [15]. A hormonal imbalance is a possible reason for the increased prevalence in females. The study found that fluctuating estrogen levels can contribute to chronic headaches or migraines [16].

This study also examined the association between hypertension status and headache in Al-Kharj. This is in line with a prospective research in Norway which found that high systolic blood pressure at baseline appeared to be associated with a low prevalence of headache in all age groups 11 years later [17]. They found an overall inverse relationship between systolic blood pressure and headaches among females but not among males in their study's cross-sectional part. A possible explanation for this is the hypalgesia-associated hypertension phenomenon that probably involves the baroreflex system, which influences nociception in the brain stem or spinal cord [17]. However, previous studies documented a substantial higher proportion of normotensive individuals (93.3%) reporting headaches compared with individuals with hypertension (6.7%) [17].

The multiple logistic regression analyses suggest that being a current smoker raised the odds of developing headaches by 32%, which indicates that smoking is associated with an increased probability of having a headache. Likewise, a study represents that current smokers have 40% more chances to develop headaches than nonsmokers (OR: 1.38; 95% CI: 1.17–1.62; *P* < 0.001). The possibility of headaches has increased significantly depending upon the increase in the number of cigarettes smoked daily and the number of years spent smoking [18]. Conversely, another study reported that their research does not support a strong causal relationship between smoking intensity and headaches [19]. Correspondingly, another study shows that an individual who is a current smoker or with a history of smoking had an association with an increased risk of migraine, but not TTH [8].

With regards to individuals with diabetes and its association with headaches, no significant association was noted in this study. Similarly, a French national cohort study found a lower risk of type2 diabetes in women with migraine. They also found a linear decrease in headache prevalence after a diabetes diagnosis. This may suggest the role of hyperglycemia and hyperinsulinism on headache occurrence [20]. Likewise, a case-control study reported that patients with diabetes are less likely to present with headache [21]. However, a research in Iraq found that headaches' prevalence is more prevalent in patients with diabetes compared to those without diabetes [22].

Individuals who were overweight showed a higher prevalence of headache compared to the obese individuals. Similarly, a Chinese study found that chronic migraine and a tension-type headache were more common in higher BMI patients [23]. Likewise, individuals being obese and having episodic headaches were five times more likely to develop chronic daily headaches than normal weight individuals [24]. Other studies also confirmed a significant association between obesity and chronic daily headache [25, 26].

The first limitation is the cross-sectional nature of the study. Second, as study was carried out in Al-Kharj and not in other areas, it may be controversial to generalize our findings to the entire population of Saudi Arabia. Despite the limitations, the characteristics of the sample are comparable to the Al-Kharj population of Saudi Arabia. The study's strength lies in the large sample that was randomly selected with wide range of age distribution and variable demographical characteristics, making it possible to identify predictors and factors of headache and measure their relationships in the Al-Kharj population.

## 5. Conclusion

The study shows that headaches are common ailment prevalent among females. In addition, smoking and overweight were significantly associated factors with headaches. The etiological existence of these relationships needs to be verified by potential prospective studies. However, the prevalence of headaches is not very high in the general adult population. Yet, future studies should also be performed, including children and more risk factors. Future studies should be performed prospectively to find out the direction of causality.

## Figures and Tables

**Table 1:** Univariate analysis regarding headache status in the Al-Kharj population sample (*n* = 1,019).

Variables	Headache pain			Total (*n* = 1019)	Chi-square (*X*^2^)	*P* value
	Yes (*n* = 30)	No (*n* = 989)				
	*n* (%)	*n* (%)				
Respondent gender						
Male	12 (40.0)		369 (37.3)	381 (37.4)	13.090	0.047
Female	18 (60.0)		620 (62.7)	638 (62.6)		

Marital status						
Not married	20 (66.7)		642 (64.9)	662 (65.0)	14.039	0.038
Married	10 (33.3)		347 (35.1)	357 (35.0)		

Education level						
Primary	0 (0.0)		22 (2.2)	22 (2.2)	1.561	0.816
Secondary	4 (13.3)		127 (12.8)	131 (12.9)		
Intermediate	1 (3.1)		29 (2.9)	30 (2.9)		
University	23 (76.7)		777 (78.6)	800 (78.5)		
Postgraduate	2 (6.7)		34 (3.4)	36 (3.5)		

Job type						
Not working	1 (3.3)		31 (3.1)	32 (3.1)	0.620	0.733
Civilian	14 (46.7)		533 (53.9)	547 (53.7)		
Soldier	15 (50.0)		524 (43.0)	440 (43.2)		

Prehypertension status						
Prehypertensive	19 (63.3)		540 (53.9)	559 (54.2)	1.034	0.309
Normotensive	11 (36.7)		461 (46.1)	472 (45.8)		

Hypertension status						
Hypertensive	2 (6.7)		48 (4.8)	50 (4.8)	9.221	0.048
Normotensive	28 (93.3)		953 (95.2)	981 (95.2)		

Diabetes status						
Nondiabetic	30 (100.0)		944 (95.4)	974 (95.6)	1.428	0.232
Diabetic	0 (0.0)		45 (1.6)	45 (4.4)		

Smoking status						
Nonsmoker	25 (83.3)		862 (87.2)	887 (87.0)	18.412	0.027
Ex-smoker	0 (0.0)		31 (3.1)	31 (3.0)		
Current smoker	5 (16.7)		96 (9.7)	101 (9.9)		

BMI class						
Nonobese (<25 kg/m^2^)	12 (40.0)		453 (45.9)	465 (45.7)	18.570	0.046
Overweight (25–29.9 kg/m^2^)	11 (36.7)		261 (26.4)	272 (26.7)		
Class I obese (30–34.9 kg/m^2^)	4 (13.3)		165 (16.7)	169 (16.6)		
Class II/III obese (≥35 kg/m^2^)	3 (10.0)		109 (11.0)	112 (11.0)		

**Table 2:** Logistic regression model using BMI (continuous variable) as a predictor for headache status (binary outcome), after adjusting for sociodemographic and other variables (*n* = 1,019).

Headache status	B	S.E. of B	*P* value	Exp (B)/Odds ratio	95% CI for odds ratio	
					Lower	Upper
Body Mass index (BMI)	1.58	0.030	0.042	1.58	1.421	1.752
Age	0.007	0.038	0.856	0.993	0.922	1.070
Gender (female)	−0.501	0.681	0.024	0.735	0.612	1.341
Marital status (married)	−0.063	0.560	0.106	0.936	0.313	2.804
Education level	0.548	1.318	0.677	1.730	0.131	10.171
Job (not working)	0.825	0.669	0.217	2.282	0.615	8.460
Job (civilian)	0.854	1.311	0.515	2.348	0.180	9.485
Diabetes (yes)	4.698	0.318	0.684	2.282	0.615	8.471
Smoking status (current smoker)	2.418	0.524	0.037	1.319	0.932	2.462

B, beta coefficient; S.E of B, standard error of beta coefficient.

**Table 3:** Logistic regression model using BMI class (categorical variable) as a predictor for headache status (binary outcome) after adjusting for sociodemographic and other variables (*n* = 1,019).

Headache status	B	S.E. of B	*P* value	Exp (B)/Odds ratio	95% CI for odds ratio	
					Lower	Upper
BMI class (overweight)	1.526	0.971	0.037	1.631	1.48	1.854
Age	0.007	0.038	0.846	0.993	0.921	1.069
Gender (female)	−0.505	0.680	0.023	0.604	0.417	1.283
Marital status (married)	−0.058	0.541	0.047	0.875	0.646	2.317
Education level	0.536	1.227	0.574	1.841	0.362	9.153
Job (not working)	0.726	0.658	0.228	2.379	0.628	8.774
Job (civilian)	0.836	1.290	0.521	2.159	0.178	7.352
Diabetes (yes)	4.587	0.329	0.476	2.317	0.716	8.372
Smoking status (current smoker)	1.306	0.539	0.138	1.289	0.528	6.871

B, beta coefficient; S.E of B, standard error of beta coefficient.

## Data Availability

The datasets generated and analyzed to support the findings of this study are provided by Prince Sattam Bin Abdulaziz University under license and are not publicly available. The datasets used to support the findings of this study are available from the corresponding author upon request, with permission of Prince Sattam Bin Abdulaziz University.
